# Vestibular rehabilitation with virtual reality in Ménière's disease

**DOI:** 10.5935/1808-8694.20130064

**Published:** 2015-10-04

**Authors:** Adriana Pontin Garcia, Mauricio Malavasi Ganança, Flávia Salvaterra Cusin, Andreza Tomaz, Fernando Freitas Ganança, Heloisa Helena Caovilla

**Affiliations:** a PhD in Sciences at the Graduate Program on Human Communication Disorders of the Federal University of São Paulo - Paulista Medical School (UNIFESP-EPM). (Professor in the Speech and Hearing Therapy Program at FMU).; b Full Professor of Otorhinolaryngology at UNIFESP-EPM.; c MSc in Sciences at the Graduate Program on Human Communication Disorders at UNIFESP-EPM; Speech and Hearing Therapist. (Professor in the Speech and Hearing Therapy at Faculdade Metropolitana Unida - FMU).; d MSc in Sciences in the Graduate Program on Human Communication Disorders at UNIFESP-EPM (PhD student in Sciences at the UNIFESP-EPM Graduate Program on Human Communication Disorders).; e Post-doctoral degree in Medicine at UNIFESP-EPM (Adjunct Professor in the Otoneurology Course in the Department of Otorhinolaryngology and Head and Neck Surgery at UNIFESP-EPM).; f Senior Associate Professor in the Course of Otology and Otoneurology at UNIFESP-EPM. Course of Otology and Otoneurology - UNIFESP-EPM.

**Keywords:** dizziness, Ménière disease, rehabilitation

## Abstract

Virtual reality technology can provide a wide range of sensory stimuli to generate conflicts of varying degrees of complexity in a safe environment.

**Objective:**

To verify the effect of a virtual reality-based balance rehabilitation program for patients with Menière's disease.

**Method:**

This observational clinical study included 44 patients aged between 18 and 60 years diagnosed with Menière's disease submitted to a controlled randomized therapeutic intervention. The case and control groups took betahistine and followed a diet. Case group subjects underwent 12 rehabilitation sessions with virtual reality stimuli in a Balance Rehabilitation Unit (BRU™). Patients were assessed based on DHI scores, the dizziness visual analogue scale, and underwent posturography with virtual reality before and after the intervention.

**Results:**

After the intervention, the case group showed significantly lower scores in DHI (*p* < 0.001) and in the dizziness visual analog scale (*p* = 0.012), and had significantly greater limit of stability areas (*p* = 0.016) than controls.

**Conclusion:**

Virtual reality-based balance rehabilitation effectively improved dizziness, quality of life, and limit of stability of patients with Menière's disease.

## INTRODUCTION

Ménière's disease is characterized by tinnitus, vertigo, and hearing loss in paroxysmal episodes without the involvement of the central nervous system. The condition was described by Prosper Ménière in 1861^1^. Endolymphatic hydrops is believed to be the pathophysiological basis of the disease. However, the specificity of the association between evidences of endolymphatic hydrops and clinical manifestations of the disease does not appear to be absolute^2^.

Ménière's disease has been described as recurring vertigo episodes lasting for a minimum of 20 minutes accompanied by nausea and vomiting, spontaneous horizontal rotational nystagmus, hearing loss, aural fullness, and tinnitus. Four levels of certainty have been attributed to the diagnosis of Ménière's: certain, when confirmation is available through pathology testing; definite, when the patient has had two or more spontaneous episodes of vertigo lasting for at least 20 minutes, sensorineural hearing loss, tinnitus or sensation of aural fullness in the involved ear; probable, when the patient has had one episode of vertigo, sensorineural hearing loss, tinnitus or aural fullness in the affected ear; and possible, when the patient has had recurrent episodic vertigo without documented hearing loss, or fluctuating or stable sensorineural hearing loss with imbalance without recurrent episodic vertigo^3^.

Vestibular rehabilitation has been proposed to improve the quality of life of individuals experiencing dizzy spells and body imbalance. It is based in a program of exercises for the eyes, head, and body, involving specific physical maneuvers associated with changes of life style e clarification on imbalance. Vestibular rehabilitation is a physiological, innocuous, coherent therapy that acts on the vestibular system to stimulate central nervous system plasticity, promote the reinstatement of body balance, accelerate and stimulate the natural mechanisms of compensation, adaptation, and acclimatization^4^.

The exercises aim to modify the subject's postural control system by exposing the patient to different visual environments along with congruent and conflicting stimuli; mitigate dizziness and body imbalance; enhance the stability of the patient's gaze and improve his/her postural control, competence, and well-being while performing activities of daily living^5^.

There is no consensus on the use of body balance exercise programs in patients with Ménière's, given the fluctuating nature of the disease. Some authors believe that the ideal candidate for body balance rehabilitation is the patient with stable Ménière's disease^6^, ^7^; others recommend exercises in two situations: 1) patients who underwent destructive therapies such as vestibular neurectomy or labyrinthectomy to accelerate the recovery of balance and postural stability^8^; and 2) patients with body imbalance persisting without the other symptoms of Ménière's^9^.

Virtual reality enables patients to dive into a world of illusion. The perception of the environment is modified by artificial stimuli, generating sensory conflict and altering the gain of the vestibulo-ocular reflex^10^. Repetitive movements of images on the retina produced by virtual reality devices designed to control visual stimuli may induce vestibular response adaptation^11^ and adjust the vestibulo-ocular and vestibulospinal reflexes involved in postural control and body balance strategies^12^, ^13^, ^14^.

Virtual reality technology enables therapists to offer patients a wide range of highly specific stimuli and sensory conflicts of varying degrees of complexity in a safe environment^15^.

Few studies have been published on the use of virtual reality in vestibular rehabilitation. Patients with Ménière's disease, when compared to healthy subjects, presented significantly greater areas of center of pressure (CoP) and higher oscillation rates in Balance Rehabilitation Unit (BRU™)^16^ posturography, indicating the usefulness of this procedure in patient assessment. Patients with central vestibular syndromes^13^and elderly subjects suffering from imbalance and risk of falls^17^ showed improved posturography parameters after treatment with virtual reality. However, stability limit areas and oscillation rates were similar before and after 12 sessions of rehabilitation with virtual reality in a pilot study in which ten subjects with Ménière's were treated twice per week^18^.

Virtual reality may be an important tool in the treatment of subjects with Ménière's - a disease with significant incidence rates, ranging from 7.7 to 157 per 100,000 people^19^, ^20^. The lack of case-control studies on the topic motivated the verification of the efficacy of this instrument in the rehabilitation of the vestibular systems of patients with the disease.

This study aimed to verify the effect of a body balance program based on stimuli produced through virtual reality in patients with Ménière's disease.

## METHOD

This randomized controlled cohort study was carried out in the Vestibular Rehabilitation Section of the Otology and Otoneurology Course administered in the Department of Otorhinolaryngology and Head and Neck Surgery of the Federal University of São Paulo - Paulista Medical School (UNIFESP-EPM) from 2008 to 2011. The study was approved by the institution's Research Ethics Committee and given permit 1142/08.

All patients included in the study were provided with clarification and asked to give informed consent.

Patients of both genders, aged between 18 and 60 years, diagnosed with definite^3^ Ménière's disease by an ENT, and with complaints of dizziness in the disease's intercritical periods were enrolled in the study.

Study participants were taking betahistine (one 24 mg dose every 12 hours) and had been under ENT follow-up. In dietary terms, they were asked to have substantial breakfasts, light lunches, and even lighter dinners; avoid intervals greater than three hours between meals; refrain from having refined sugar, coffee, or alcohol; and refrain from smoking.

Patients diagnosed with bouts of the disease by the ENT physician immediately before the beginning of the study were excluded, as were subjects with rheumatic diseases, uncontrolled high blood pressure, heart disease, severe visual involvement or decompensated involvement despite contact lenses, orthopedic disorders resulting in motion limitation or use of lower limb prostheses, psychiatric disorders, individuals submitted to stem cell transplant, patients unable to comprehend and obey simple verbal commands or stand independently in an orthostatic position, subjects who drank alcohol 24 hours before the tests, patients submitted to balance rehabilitation programs in the six months prior to the study, subjects who missed three consecutive body balance rehabilitation sessions, and those who failed to follow the orientations proposed by the authors of the study.

The otoneurological assessment of the enrolled patients included interviews, the Dizziness Handicap Inventory (DHI) to assess quality of life, the analog dizziness scale, ENT examination, pure-tone audiometry, speech intelligibility testing, impedance testing, functional vestibular examination, and posturography with virtual reality in the Balance Rehabilitation Unit (BRU™). DHI, dizziness analog scale, and posturography tests were performed before and after the intervention.

The DHI's^21^ Brazilian Portuguese version^22^ was used to evaluate the self-perceived incapacitating effects of dizziness before and after the completion of vestibular rehabilitation with virtual reality. Relevant improvements were noted when the difference between the DHI scores before and after the intervention was greater than 18 points^21^. Self-perceived dizziness in DHI score analysis was categorized as mild (scores between zero and 30), moderate (31 to 60), and severe (61 to 100)^23^.

The dizziness analog scale^24^ was used to verify symptom intensity before and after the intervention. Scores may range between zero, reflecting the lowest level of dizziness, and ten, in reference to the highest levels of dizziness.

BRU^™25^ was used to assess and rehabilitate patients with dizziness and associated symptoms by providing them with visual stimuli projected in virtual reality goggles. The equipment includes a computer with the test's program, a safety metal frame, protection support with straps and belts, a force platform measuring 40 x 40 cm, virtual reality goggles, an accelerometer and foam cushions. Three modules were included: posturography, body balance rehabilitation, and postural training games (PTG). The equipment was set up in a silent room of approximately six square meters with dim lighting^26^.

The BRU™ posturography module provides information on the position of the patient's center of pressure by measuring the stability limit area, CoP area, body oscillation rate in ten sensory conditions with the subject in an orthostatic position: 1) with open eyes; 2) with eyes closed; 3) with closed eyes on a compliant surface; 4) saccadic stimulation; 5) optokinetic stimulation in the horizontal direction from left to right; 6) optokinetic stimulation in the horizontal direction from right to left; 7) optokinetic stimulation in the vertical direction looking up and down; 8) optokinetic stimulation in the vertical direction looking down and up; 9) optokinetic stimulation in the horizontal direction associated with slow steady rotation of the head; 10) optokinetic stimulation in the vertical direction associated with slow steady flexion and extension movements of the neck^25^.

In order to assess each of the ten sensory conditions, patients were instructed to stand in an orthostatic position without moving their upper limbs, heels, or feet for 60 seconds. They were allowed to wear their usual corrective lenses. A foam cushion of medium density was used in the third test condition. Virtual reality goggles were used from the fourth to the tenth condition. Patients were allowed to rest during the procedure as needed. Patients wore safety straps and belts and the examiner stood close to them to prevent the occurrence of falls during the tests.

The program generated reports containing data on stability limit areas, CoP areas (95% confidence ellipse), and oscillation rates in the ten test conditions. The 95% confidence ellipse was defined as the area in which 95% of the center of pressure points were captured during the test. Oscillation rates were calculated by the total distance divided by the 60 seconds of the test's duration^25^.

The 44 patients diagnosed with unilateral or bilateral definite Ménière's disease were divided into case and control groups according to a table with uniformly distributed random numbers produced by a computer program.

The randomization process assigned 23 patients to the case group and 21 to the control group. Unilateral disease was seen in 22 of the 23 patients in the case group and in 21 controls. Seven patients in the case group had unilateral vestibular hypofunction; one had directional preponderance nystagmus; fourteen had normal vestibular function; and one never completed the tests due to intense neurovegetative symptoms Eight patients in the control group had unilateral vestibular hypofunction, and 13 had normal vestibular function.

Subjects in the control group were given dietary recommendations and prescribed 48 mg/day of betahistine (one 24 mg dose every 12 hours). In addition to a similar diet and drug therapy, case group individuals performed stimulus-enriched exercises on the BRU™. All patients were evaluated as soon as a diagnosis of definite Ménière's disease was rendered by an ENT physician. Case group subjects were reassessed immediately after the end of the intervention, while controls were seen six weeks after treatment.

Body balance rehabilitation exercises were performed at the clinic twice a week with each patient, adding to a total of 12 sessions. Each session lasted 45 minutes and was planned based on the sensory conditions and altered postural parameters seen in the posturography of each individual.

The balance rehabilitation module in the BRU™ was made up of a virtual image emitter and 3D goggles to create situations that triggered dizzy spells or vertigo episodes or aided in the compensation of vestibular disorders^17^. Body balance rehabilitation included visual and somatosensory stimuli and the PTG™ module in the BRU™, in three interactive training games on postural control, stability limit, and muscle coordination covering various motor tasks in varying degrees of difficulty. All patients were exposed to foveal (smooth pursuit and saccades), retinal (bars, tunnel, and optokinetic train) and sensory integration (vestibulo-ocular reflex, suppression of the vestibulo-ocular reflex, vestibular optokinetic reflex) visual stimuli. Patient skill level and evolution aided in the setting up of the visual stimuli in terms of latency, duration, frequency, motion, and depth, in addition to serving as input on the progression of somatosensory stimuli and changes such as the surface patients had to stand on during the tests, from firm pads to foam pads of varying density; walking on the spot on a firm and a compliant surface; and bouncing on a swiss ball. Postural control improvements were observed when significant increases on stability limit values and significant reductions on CoP area and BRU™ oscillation rates were seen after the intervention.

Patients were informed of all treatment phases and of the occurrence of dizzy spells during the exercises, particularly in the early sessions. They were also made aware of the importance of complying with the exercise regimen.

The evaluations and the rehabilitation program were carried out by the head researcher. After the intervention, the patients were referred to an ENT physician for advice on the continuation of the treatment.

Results were specified, treated, and submitted to statistical analysis on software program SPSS (Statistical Package for Social Sciences) version 19.0. A level of significance of 5% (α = 0.05%) was adopted. In the description of the sample, categorical variables were characterized in terms of frequencies and their respective percentages, while scalar variables were presented in the form of the following frequency calculations (n): mean values, standard deviations, minimum and maximum values. Comparisons between case and control groups were performed using Fisher's exact test for categorical variables, and Mann-Whitney's test to look into the interdependences between sample elements of a scalar nature. In-group comparisons before and after intervention were carried out using Wilcoxon's test.

## RESULTS

Table 1 shows the distribution of the 44 patients diagnosed with definite Ménière's according to age, gender, and duration, periodicity and time since the onset of dizzy spells. No statistically significant differences were found between the groups in terms of age, gender, and duration, periodicity or time since onset of dizzy spells.

**Table 1:** Demographic data and clinical characteristics of the patients in the case and control groups before the intervention.

Variable	Case group (n = 23)	Control group (n = 21)	*p*
Mean age (minimum-maximum)	47.65 (20-60)	47.90 (19-60)	0.869^a^
Gender (male/female), n (%)	9 (39.10)/14 (60.90)	7 (33.30)/14 (66.70)	0.761^b^

Duration of dizzy spells, n (%)			

Days	9 (39.10)	7 (33.33)	
Hours	8 (34.80)	7 (33.33)	0.709^b^
Minutes	5 (21.70)	4 (19.01)	
Seconds	1 (4.30)	3 (14.33)	

Periodicity of dizzy spells, n (%)			

Sporadic	5 (21.70)	12 (57.10)	
Monthly	4 (17.40)	1 (4.80)	0.087^b^
Weekly	8 (34.80)	4 (19.00)	
Daily	6 (26.10)	4 (19.00)	

Time since onset of dizzy spells, n (%)			

3 to 6 months	1 (4.30)	2 (9.50)	
7 to 11 months	1 (4.30)	1 (4.80)	
1 to 2 years	4 (17.40)	6 (28.60)	0.108^b^
2 to 4 years	5 (21.70)	0 (0.00)	
More than 5 years	12 (52.20)	12 (57.10)	

a Mann-Whitney test;

b Fisher's exact test.

Table 2 presents comparisons including DHI scores, dizziness analog scale scores, and stability limit areas before and after the intervention, featuring both in-group and between group analyses. The comparison of case and control groups before the intervention failed to reveal significant differences on DHI scores, dizziness analog scale scores, or stability limit areas. After the intervention, DHI and dizziness analog scale scores were significantly lower, whereas stability limit areas were significantly larger among case group subjects. Among controls, the dizziness analog scale had significantly lower scores.

**Table 2:** Comparison between case and control groups before and after the intervention: Dizziness Handicap Inventory, dizziness analog scale, and stability limit area.

		Case Group			Control Group			
Tests	Before Mean/SD (min-max)	After Mean/SD (min-max)	p1	Before Mean/SD (min-max)	After Mean/SD (min-max)	p1	p2	p3
DHI (total)	57.57/21.27 (12.00-94.00)	22.87/22.07 (0.00-72.00)	< 0.001*	52.67/21.39 (18.00-86.00)	48.38/22.37 (8.00-86.00)	0.092	0.391	< 0.001*
DHI (physical)	17.04/7.26 (2.00-28.00)	6.61/6.91 (0.00-24.00)	< 0.001*	15.62/6.25 (4.00-32.00)	13.33/7.98 (0.00-32.00)	0.075	0.24	0.003*
DHI (functional)	22.78/10.00 (4.00-40.00)	9.48/8.64 (0.00-32.00)	< 0.001*	18.67/9.11 (6.00-40.00)	17.33/8.45 (2.00-28.00)	0.274	0.131	0.004*
DHI (emotional)	17.91/8.64 (0.00-32.00)	6.78/8.20 (0.00-26.00)	< 0.001*	18.38/8.76 (4.00-32.00)	17.33/9.81 (0.00-32.00)	0.447	0.962	0.001*
Dizziness analog scale	7.17/2.06 (3.00-10.00)	2.57/2.41 (0.00-7.00)	< 0.001*	7.81 / 2.16 (4.00-10.00)	5.43/4.58 (1.00-22.00)	0.009*	0.283	0.012*
Stability limit	222.22/66.16 (70.00-365.00)	236.22/62.41 (70.00-373.00)	< 0.001*	185.24/52.85 (88.00-307.00	190.10/60.86 (78.00-286.00)	0.566	0.307	0.016*

Wilcoxon test;

Mann-Whitney test; DHI: Dizziness Handicap Inventory; SD: Standard deviation; Min: Minimum value; Max: Maximum value;

*p*1 : In-group comparison;

*p*2 : Comparison between groups before intervention;

*p*3 : Comparison between groups after intervention;

* Statistically significant difference.

Table 3 draws comparisons of CoP areas seen in BRU™ posturography before and after the intervention between case group subjects, controls, and both groups. When case and control groups were considered, the comparison between the values for CoP area in the ten tested sensory conditions after the intervention failed to yield significant differences. After the intervention, case group subject CoP areas in the firm surface with eyes closed, and compliant surface with eyes closed conditions were significantly smaller. No significant differences were seen among controls for CoP area in the ten tested sensory conditions.

**Table 3:** In-group comparisons of center of pressure areas (cm^2^) before and after intervention.

		Case Group			Control Group			
Conditions	Before Mean/SD (min-max)	After Mean/SD (min-max)	p1	Before Mean/SD (min-max)	After Mean/SD (min-max)	p1	p2	p3
FS/open eyes	4.62/7.68 (0.15-33.65)	3.16/4.08 (0.44-19.04)	0.362	5.04/12.05 (0.33-56.85)	2.67/4.96 (0.71-23.86)	0.095	0.851	0.496
FS/eyes closed	5.50/8.89 (0.21-38.93)	2.84/313 (0.18-12.16)	0.026*	4.53/9.75 (0.94-1.27)	4.69/10.06 (0.21-47.70)	0.211	0.359	0.597
CS/eyes closed	13.98/19.76 (1.63-95.37)	7.92/5.71 (0.99-25.59)	0.042*	13.00/14.95 (2.04-53.59)	12.72/20.20 (1.63-95.43)	0.664	0.888	0.842
FS/saccades	1.74/1.44 (0.19-4.46)	2.07/1.56 (0.15-7.30)	0.574	2.33/3.76 (0.27-18.18)	3.10/7.78 (0.26-14.78)	0.889	0.706	0.307
FS/optokinetic, right	2.62/2.82 (0.28-10.70)	3.10/3.98 (0.26-14.78)	0.784	4.48/10.40 (0.34-47.94)	6.97/20.72 (028-96.35)	0.414	0.751	0.991
FS/optokinetic, left	4.93/8.07 (0.11-26.18)	2.80/3.26 (0.09-13.52)	0.171	5.59/15.78 (0.26-73.21)	6.07/16.09 (0.21-74.96)	0.052	0.751	0.751
FS/optokinetic, down	2.80/2.84 (0.15-11.09)	2.56/3.28 (0.11-16.13)	0.475	3.69/7.08 (0.23-33.15)	5.75/15.76 (0.18-73.32)	0.931	0.953	0.916
FS/optokinetic, up	4.16/5.96 (0.12-26.20)	3.35/5.66 (0.12-24.23)	0.386	4.38/7.83 (0.33-34.69)	4.86/11.85 (0.12-24.23)	0.728	0.897	0.605
FS/optokinetic, horizontal	4.81/6.97 (0.69-33.70)	3.43/5.70 (0.42-28.91)	0.200	4.84/6.68 (0.45-28.74)	5.62/12.71 (069-60.39)	0.689	0.796	0.459
FS/optokinetic, vertical	4.14/6.52 (0.76-32.11)	3.24/4.02 (0.52-20.21)	0.922	4.10/5.22 (0.53-24.46)	5.44/12.07 (0.64-57.57)	0.768	0.597	0.445

Wilcoxon test;

Mann-Whitney test; FS: Firm surface; CS: Compliant surface; SD: Standard deviation; Min: Minimum value; Max: Maximum value;

*p*1 : In-group comparison;

*p*2 : Comparison between groups before intervention;

*p*3 : Comparison between groups after intervention;

* Statistically significant difference.

Table 4 displays a comparison of the oscillation rates in BRU™ posturography before and after the intervention between case group subjects, controls, and both groups. No statistically significant differences were seen in the comparison of oscillation rates in the ten tested sensory conditions between case and control groups before and after intervention. After the intervention, case group subjects showed significantly lower oscillation rates in the compliant surface with eyes closed condition and significantly higher oscillation rates in conditions of saccade stimulation and optokinetic stimulation in the vertical direction. Oscillation rates were not significantly different between controls in the ten tested sensory conditions.

**Table 4:** Comparison between case and control groups before and after intervention; oscillation rates (cm/s^2^).

		Case Group			Control Group			
Conditions	Before Mean/SD (min-max)	After Mean/SD(min-max)	p1	Before Mean/SD (min-max)	After Mean/SD(min-max)	p1	p2	p3
FS/open eyes	0.85/0.52 (0.39-2.88)	0.84/0.57 (0.43-3.16)	0.976	0.92/1.00 (0.36-4.42)	0.92/0.98 (0.39-5.06)	0.289	0.431	> 0.999
FS/eyes closed	1.11/0.70 (0.45-3.75)	1.02/0.70 (0.39-3.69)	0.173	1.16/1.05 (0.44-4.99)	1.39-1.83 (0.42-9.07)	0.144	0.488	0.526
CS/eyes closed	2.68/1.69 (1.33-9.69)	2.22/1.06 (0.95-5.23)	0.021	2.60/1.36 (1.23-9.69)	2.61/1.68 (1.00-7.90)	0.917	0.888	0.445
FS/saccades	0.95/0.56 (0.47-3.26)	1.09/0.59 (0.45-3.10)	0.012*	0.94/0.50 (0.49-2.87)	1.16/1.36 (0.46-7.01)	0.525	0.832	0.589
FS/optokinetic, right	0.93/0.32 (0.48-1.73)	1.14/1.04 (0.44-5.50)	0.773	1.08/1.20 (0.51-6.24)	1.43/2.55 (0.52-12.48)	0.104	0.438	0.934
FS/optokinetic, left	1.04/0.57 (0.41-3.07)	1.06/0.62 (0.40-3.02)	0.910	1.00/0.86 (0.37-4.62)	1.47/2.76 (0.48-13.49)	0.211	0.347	0.944
FS/optokinetic, down	0.93/0.44 (0.39-2.46)	1.04/0.62 (0.41-2.96)	0.294	0.96/0.71 (0.38-3.93)	1.35/2.28 (0.42-11.23)	0.273	0.630	0.934
FS/optokinetic, up	1.04/0.44 (0.37-2.12)	1.12/0.64 (0.46-3.10)	0.495	1.07/0.97 (0.48-5.13)	1.29/1.88 (0.37-9.38)	0.543	0.347	0.488
FS/optokinetic, horizontal	1.34/0.66 (0.69-3.87)	1.67/1.88 (0.76-10.00)	0.412	1.38/0.94 (0.59-4.90)	1.69/2.36 (0.56-11.80)	0.931	0.638	0.597
FS/optokinetic, vertical	1.43/0.70 (0.78-3.54)	1.77/1.42 (0.89-7.84)	0.039*	1.52/0.98 (0.81-5.30)	1.83/2.54 (0.42-12.68)	0.903	0.733	0.209

Wilcoxon test;

Mann-Whitney test; FS: Firm surface; CS: Compliant surface; SD: Standard deviation; Min: Minimum value; Max: Maximum value;

*p*1 : In-group comparison;

*p*2 : Comparison between groups before intervention;

*p*3 : Comparison between groups after intervention;

* Statistically significant difference.

## DISCUSSION

This study looked into the effects of vestibular rehabilitation using virtual reality on patients with Ménière's disease. The 21 subjects in the control group were given dietary recommendations and prescribed 48 mg/day of betahistine (one 24-mg dose every 12 hours), while the 23 individuals in the case group, in addition to similar diets and drug therapy, were asked to perform exercises with visual, proprioceptive, and vestibular stimulation on the BRU™*.* We were unable to find other studies in the literature with the same therapeutic design or in which results had been compared against a control group.

In the initial assessment before the intervention, patients in the case and control groups were similar in terms of gender, age, and duration, periodicity, and time since the onset of of dizzy spells; DHI scores; dizziness analog scale scores; vestibular examination findings; limits of stability; CoP area and oscillation rates in all BRU™ posturography conditions, indicating the sample was homogeneous. DHI indicated moderate impact of symptoms upon quality of life^23^ in case group subjects (mean score of 57.57) and controls (mean score of 52.67) before treatment. However, another study found mild impact (mean score of 31.00) of symptoms upon the quality of life^23^ of patients with Ménière's disease^27^.

After the intervention, controls showed reduced scores in the dizziness analog scale, although not accompanied by reduced DHI scores or larger stability limit areas. There are no papers in the literature describing the effects of BRU™ rehabilitation on patients with Ménière's taking betahistine and submitted to dietary orientation.

Case group subjects improved from dizziness and reported better quality of life after the intervention, as interpreted from the reduced scores in the dizziness analog scale and the difference of over 18 points in the DHI scores. Increases in the stability limit areas suggested improved ability to maintain body balance while moving without altering the support base. Similarly to our findings, patients with Ménière's also reported improved quality of life after vestibular rehabilitation associated with psychotherapy^28^, after taking betahistine^29^, and after undergoing rehabilitation on a BRU^™18^. Improvements on the intensity of the dizzy spells were also observed^18^. Patients with Ménière's disease submitted exclusively to treatment on a BRU™ failed to show significant increases on stability limits^18^, possibly due to the small size of the sample. No papers in the literature were found to describe the effects of BRU™ rehabilitation on patients with Ménière's taking betahistine and submitted to dietary orientation in relation to DHI scores, the dizziness analog scale, and stability limit values.

After rehabilitation aided by virtual reality resources, patients in the case group performed differently from controls in terms of total DHI scores and physical, functional, and emotional aspects. Differences were also seen in dizziness analog scale scores and stability limit areas, with case group individuals presenting more significant score reductions and, therefore, improvements from dizzy spells and in terms of quality of life when compared against controls. The significant increase in stability limit areas showed that case control subjects were better at moving and maintaining balance without altering the support base. No papers in the literature were found to compare the efficacy of BRU™ rehabilitation on patients with Ménière's taking betahistine and submitted to dietary orientation against controls taking betahistine and submitted to dietary orientation.

After the intervention, the performance of case group subjects was the same as found in controls in regards to CoP area values. However, case group individuals had smaller CoP areas when tested on a firm surface with eyes closed and on a compliant surface with eyes closed after the intervention, a finding that may be seen as a favorable effect of the rehabilitation program aided by virtual reality. Ten patients with Ménière's disease submitted exclusively to BRU™ rehabilitation failed to show significant reductions on CoP areas in the ten sensory conditions in which they were tested^18^. Patients with central vestibular disorders^13^and elderly patients with instability and risk of falls^17^ showed reduced CoP areas in a firm surface with open eyes and during optokinetic stimulation to the right and to the left after vestibular rehabilitation with virtual reality.

After the intervention, the performance of the case group was similar to that of the control group in terms of oscillation rates, except for when subjects were tested on a compliant surface with eyes closed, which may be considered as a favorable effect provided by rehabilitation with virtual reality and of the increases seen during saccade and optokinetic stimulation in the vertical direction, possibly meaning that rehabilitation could not favorably interfere with the evolution of oscillation rates in these two conditions. As also seen in this study, patients with Ménière's submitted to BRU™ rehabilitation had significant increases in oscillation rates in optokinetic stimulation in the vertical direction^18^. After vestibular rehabilitation with virtual reality and analysis of three sensory conditions (not the ten sensory conditions assessed with the BRU™), reductions on oscillation rates when patients with central vestibular disorders^13^ and elderly subjects with instability and risk of falls^17^ were tested on a firm surface with open eyes and on optokinetic stimulation to the right and to the left.

No assessment instrument was able to consider all aspects involved in body balance. Today, body balance can be clinically verified through quality-of-life questionnaires, functional testing, and posturography. The procedures used to assess imbalance must be selected based on the goal of the assessment and the type of disorder observed^30^.

A quality-of-life questionnaire, the dizziness analog scale, and a posturography device were picked to assess body balance before and after the intervention. The posturography equipment was also used in the rehabilitation of patients with Ménière's disease. In the context of assessment after intervention, case group subjects may have had better outcomes than controls because they were more familiarized and trained on how to use the equipment. However, it should be pointed that only only two of the ten sensory test conditions - firm surface with eyes closed and compliant surface with eyes closed - yielded significant differences. Although posturography was not the ideal method to assess improvements after rehabilitation, we must point out that the apparent improvements resulting from stimulation in the BRU™ agreed with improvements on DHI and dizziness analog scale scores in the comparisons between case and control groups.

The visual, vestibular, and proprioceptive conflicts generated by virtual reality stimulation with the BRU™, in combination with the administration of betahistine and nutritional orientation, contributed significantly to the attainment of improvements in dizziness, quality of life, and postural control in patients with Ménière's disease.

Further studies on the use of rehabilitation aided by virtual reality in patients with Ménière's disease are required to verify how effectively other body balance functional tests can be used in the short and long terms, particularly due to the fluctuating nature of this disease.

## CONCLUSION

Body balance rehabilitation with virtual reality stimuli effectively improved symptoms of dizziness, quality of life, and stability limits of patients with Ménière's disease.
